# Empowerment model for nurse leaders’ participation in health policy development: an east African perspective

**DOI:** 10.1186/s12912-015-0078-6

**Published:** 2015-05-13

**Authors:** Nilufa Jivraj Shariff

**Affiliations:** School of Nursing and Midwifery, Aga Khan University, Kenya, Box 39340-00623, Nairobi, Kenya

**Keywords:** Empowerment, Model, Leadership, Nurse leaders, Participation

## Abstract

**Background:**

Nurses comprise the largest portion of the health care workforce in most countries; they interact closely with patients and communities, they work throughout the day and within all sectors of health care. Their breath of practice gives them a broad understanding of requirements of the health care system, of how factors in the environment affect the health outcomes of clients and communities. Nurses’ involvement in health policy development ensures that health services are: safe, effective, available and inexpensive.

**Methods:**

A Delphi survey was utilized and included the following criteria: expert panelists, three iterative rounds, qualitative and quantitative analysis, and building consensus. The overall aim of the study was to develop “An Empowerment Model for Nurse Leaders’ participation in Health Policy Development”. The study included purposively selected sample of national nurse leaders from the three East African countries of Kenya, Tanzania and Uganda. The study was conducted in three iterative rounds. Data collection tools were questionnaires. Data analysis was done by examining the data for the most commonly occurring concepts in the first round and descriptive statistics in the second and third rounds.

**Results:**

The findings of the study support the development of the “Empowerment Model for Nurse Leaders’ Participation in Health Policy Development”. Further the study identified that there was a significant gap in and barriers to participation in health policy activity and that an opportunity seems to exist to enable and develop nurse leaders’ role and involvement in this respect. There was consensus on factors considered to be facilitators and barriers to nurse leaders’ involvement in health policy development. Furthermore, consensus was achieved on essential leadership attributes that enhance nurse leaders’ participation in health policy development. The model was validated a small sample of the nurse leaders’ who participated in the study.

**Conclusion:**

The model provides a framework with an aim of facilitating involvement in health policy activity. Nurses need to be strategic in ensuring that they place themselves and others on the forefront of the policy development arena. The empowerment model suggests proactive and strategic involvement of nurses and nurse leaders in health policy development activities.

**Electronic supplementary material:**

The online version of this article (doi:10.1186/s12912-015-0078-6) contains supplementary material, which is available to authorized users.

## Background

Nurses comprise the largest portion of the health care workforce in most countries; they interact closely with patients and communities, they work throughout the day and night, and within all sectors of health care [1]. Their breadth of practice gives them a broad understanding of requirements of the health care system, of how factors in the environment affect the health outcomes of clients and communities and of how people respond to various strategies and services [1]. The International Council of Nurses (ICN) has long adopted the position that nurses have an important contribution to make in health service planning and decision-making and in the development of appropriate and effective health policy. It is imperative for nurses to participate in the creation of public policy pertaining to the determinants of health including Millennium Development Goals (MDGs) [2]. It has been recognized that in order to achieve the Millennium Development Goals, there is need for nurses’ input in health policy [3].

In studies conducted in Botswana and Kenya, researchers found that nurses’ role in the policy development process typically is limited to policy implementation [4–6]. Literature indicates that where nurses have been able to influence health policy development, there have been positive benefits for health care, patients and community, and nurses [7]. A model of engaging Nurse Leaders in health policy development has the potential to influence achievement of the MDGs and improve the health indicators of the region. Thus, they could influence access to health care and health professionals, quality, equity, and the cost of delivering health care services to patients and community [8].

In this paper empowerment is presented as a theoretical construct to guide nurses to increase levels of activity and influence in the policy development arena. Furthermore, empowerment is viewed as a continuum, a process that evolves towards increased growth and advancement [9]. In this context, empowerment is defined as support towards enabling individuals and groups to participate in actions and decision making related to health policy development denoting an influence relationship as opposed to a need for power from others [10]. This type of enablement and influence can lead to gaining control to be able to exercise one’s influence and authority to make and participate in decisions [11], and can be considered in relation to both individual and collective action. Jones, O’Toole, Hoa, Chau and Muc [12] suggest that recognizing and understanding the barriers to a goal, and identifying appropriate resources to resolve it, lead to a sense of strength for health, growth and professional development. In the context of this paper, the focus on empowerment relates to building, developing and supporting nurse leaders to participate and exercise influence in actions and decisions relating to health policy development, along a continuum of advancing expertise.

Ellenfsen, Polit and Hamilton suggest that three organizational structures which can support individuals are opportunity, power and relative numbers [13]. Participation in decision- making and policy development requires verbal and behavioral empowerment. Ryles [14] suggests that true empowerment can only be achieved when there is a balance of power between the oppressors and oppressed. Nursing is often perceived, and has often viewed itself, as an oppressed group in relation to other health care professionals and within health care structures. Indeed, Johns [15] contended that, “at the level of silence, nurses have no voice; voice is muted in the presence of more powerful others, fashioned and reinforced through self-perceived patterns of hierarchical communication and internalized threat of sanction”. In the literature reviewed empowerment has been explored from the context of the hospital setting, and practice environment at organizational level [16–18]. There is scant literature that explores or provides support at macro level of leadership empowerment. Furthermore literature from East Africa related to the notion of empowerment was limited.

In the East Africa context, nurses have not capitalized on the notions of relative numbers, power or opportunity to empower the profession. This empowerment model aims to provide a framework for enhancement of nurse leaders’ participation at health policy development. The proposed empowerment model was conceptualized and developed following a study that explored: the extent of national nurse leaders’ participation in policy development activity [6] identified consensus on factors that are barriers or facilitators to their participation [19]; and built consensus on leadership attributes necessary for participation in health policy development [20]. The intent of this paper is not present a detailed account of the results of the study as they have been presented elsewhere [6, 19–21] but to present a model that was conceptualised and validated as a framework to support and enhance nurse leaders’ participation in health policy development.

The overall aim of the study was to build an empowerment model to enhance Nurse Leaders’ participation in health policy development by: exploring the extent of nurse leaders’ participation in health policy development in East Africa; building consensus on leadership attributes necessary for nurse leaders’ participation in health policy development in East Africa; building consensus on factors that act as facilitators and barriers to nurse leaders’ participation in health policy development in East Africa.

## Methods

The Delphi approach was applied based on the following principles: panel of experts; iterative rounds, statistical analysis, and consensus building. The Delphi technique is a process of collecting group opinion on a topic of interest. It is based on the premise that ‘pooled intelligence’ enhances individual judgment and captures the collective opinion of experts [22]. It provides an opportunity for experts (panelists) to communicate their opinions and knowledge anonymously about a complex problem or a topic of interest, to see how their evaluation of the issue aligns with others, and to change their opinion, if desired, after reconsideration of the findings of the group’s work [23]. The Delphi technique was utilized as it is a useful method for developing conceptual frameworks and models [24]. National Nurse Leaders’ (expert panelists) were drawn from Kenya, Tanzania and Uganda who were in leadership positions that included national nursing professional associations, nursing regulatory bodies, ministry of health and universities. Purposive sampling was used because the intention was to include participants who were knowledgeable about the subject being studied. Ethical approval was received from University of South African (UNISA), National Ethics boards of Kenya, Tanzania and Uganda. Data were collected with questionnaires that were developed by the researcher for the study. The study was organized in three iterative rounds.

*Face validity* was achieved by pre-testing the tool for exhibiting: clarity of content, being reflective of the topic studied, clarity of language, being unambiguous and readable. *Content validity* was enhanced in three respects. First, questionnaires were developed with reference to the literature related to health policy development and nurses’ participation in health policy development. Second, all three questionnaires were pre-tested with a representative sample of nurse leaders, to ensure that the concepts included in the study were actually related to health policy development process. Third, the purposive study sample was comprised of a panel of experts who participate in the health policy development process.

The data collection tools were developed by the researcher and were questionnaires. The first round of the study posed open ended questions that were developed with reference to research literature, the second and third rounds, posed questions that were generated from concepts identified in the data obtained from the first round and were posed as close ended questions. The questionnaires were administered through emails; hand delivered and posted. The Expert Panelists were informed of the study; the risks involved; and that participation was voluntary; and return of completed questionnaire was considered consent to participate.

A purposive sample of Seventy-eight (78) expert panelists were invited to participate for the first round of the study; 37 (47 %) participated; of these thirty seven (37) invited to participate in the second round, 24 (65 %) participated, all twenty four (24) who were invited participate in the third round (100 %) participated. Expert panelist were defined as: Registered Nurses; working in a senior leadership/management capacity in East Africa; working at national/provincial (regional) level or university; due to the nature of their position/work, had exposure to participating in health policy development at provincial, national, regional or global level.

Data analysis for the first round was done by examining the qualitative data for the most commonly occurring concepts. The data was validated by independent research assistant. The second and third questionnaires were based on the concepts generated and developed into quantitative questions. The second and third rounds were analyzed with the aid of the SPSS version 15; thereafter, descriptive statistics were examined that included percentage average, standard deviation and mean.

Consensus was accepted for the second round at ≥90 %, with the rationale to ensure that the most critical concepts were retained from the survey, whereas consensus for the third round was set at ≥70 %, with the rationale to ensure that of the critical issues identified, the important issues were retained.

## Results

The demographic data indicated that the majority of the expert panelists who participated in this study were from Kenya where 30 (38 %) were invited and 16 (43.2 %) participated, followed by Tanzania where 34 (44 %) were invited and 15 (40.6 %) participated, and followed by Uganda where 14 (18 %) were invited and 6 (16.2 %) participated. Majority of the expert panelists were above 40 years of age 33 (89 %) and were female 23 (62 %). Seventy percent (26) of the expert panelists possessed a university degree. The majority of the expert panel members had considerable experience in the nursing profession 32 (86 %) had >15 years, but less so in their current position 27 (73 %) had ≤5 years of experience, which was a policy related position [19–21].

The results of the study indicated that nurse leaders’ involvement in health policy development activity appeared to be limited, inconsistent, on ad hoc ‘as opportunity presented’ basis and not available to every nurse leader (Additional file 1). Their input was enhanced at the implementation stage but limited during the other stages of the policy development process [6]. There was consensus on factors considered to be facilitators to nurse leaders’ involvement in health policy development. These facilitators included: (a) being involved; (b) being knowledgeable and skilled, (c) being supported; (d) positive image of nursing; (e) enabling structures and (f) available resources [19] (see Additional file 1). Significant barriers to nurse leaders’ participation were identified as: (a) lack of involvement; (b) lack of knowledge and skills; (c) negative image of nursing; (d) lack of enabling structures and (e) lack of resources. [19] (see Additional file 1). There was consensus on essential leadership attributes that enhance nurse leaders’ participation in health policy development [20]. These attributes include the ability to: (a) influence; (b) communicate effectively; (c) build relationships; (d) feel empowered and (e) demonstrate professional credibility [20]. An outline of the questionnaire is added as Additional file 1.

The findings of the study identified that there was a significant gap in and barriers to participation in health policy activity and that an opportunity seems to exist to enable and develop nurse leaders’ role and involvement in this respect. The impression gained from the study findings was that nurse leaders would like to be part of the policy development process and therefore empowering them to participate would increase their input in the process. The proposed model described in this paper was developed from the results of the study, with particular attention to results that represented a high degree of consensus among study participants, which are presented as support for concepts presented. The empowerment model proposes to support nurses and nurse leaders by providing a framework that would progressively assist them gain control and exercise their influence and authority to participate in health policy decisions. The model was validated by a sample of the panel of experts, who participated in all three rounds of the study.

## Empowerment model

### Aim of the model

The author proposes a theoretical model that provides a framework that can be used by nurses and nurse leaders to structure their development in building capacity and competence in policy development activity, particularly in the areas of concern to nursing and health care. It is envisaged that when nurses do so, their influence on policies will impact positively on the health of the community and population.

### Type of model

The proposed model is prescriptive in identifying a structure that is expected to foster change towards empowerment, with the resultant outcome of nurse leaders participating in health policy development activities.

### Assumptions of the model

1. Nurse leaders need to:
  - Be involved in the health policy development process and can make important contributions at the micro, meso and macro levels
  - Be empowered in order to support participation in health policy development
  - Gain competencies to participate in health policy development and need to be supported to do so through: Knowledge, Experience, Environment and Participation
  - Be proactive in seeking opportunities that will enhance their participation in health policy development
2. Nurse leaders can be empowered to participate in policy development by:
  - Equipping nurses with knowledge about health policy, leadership; politics; and political processes through educational institutions.
  - Gaining experience in health policy development through professional nursing associations and work organizations
  - Influencing the creation of an enabling environment that can facilitate their participation in health policy development
  - Participating in the health policy development process utilizing evidence based information, to generate expertise, and be visible and play their rightful role as policy makers.
  - Acquiring education that generates interest and motivates nurses’ participation in the policy development process.
  - Developing leadership attributes that support participation in health policy development

## Validation of the model

After the model was conceptualized, it was presented for validation to a sample of four expert panelists who had participated in all three Delphi rounds of the study. The aim was to provide expert validation of the model in terms of its applicability and usefulness as an empowerment model. An evaluation tool was utilized for this process. The tool was developed by the researcher with reference to literature [25–29]. It included eight criteria which were drawn up to substantiate the model as summarized in Table 1. They included: importance; precision and clarity; parsimony and simplicity; comprehensiveness; operationality; logical structure; validity; and practicality. Definitions of the validation criteria were developed with reference to literature [26–29]. A Likert scale, which included the measures for evaluating the expert panelists’ views, was drawn up: the points included accepted, accepted with minimum changes and not accepted. The feedback of the expert panelists is summarized in Table 2. They recommended minor modifications that were incorporated.

**Table 1:** Definitions of evaluation criteria

Importance:	Relates to the quality of having significance to the profession; acceptance by competent professionals may be indicative of importance [22, 25].
Precision and clarity:	Relates to being clear and whether the areas discussed in the model are consistent; this further relates to whether the descriptions of the assumptions, concepts and principles are clearly formulated [23–25].
Parsimony and simplicity:	Signify being simple and uncomplicated [25].
Comprehensiveness:	Relates to covering the broad and substantive areas of Interest [25].
Operationality:	Relates to being specific enough to be testable and measurable [25].
Logical structure:	Relates to the degree with which the visual diagram represents and simplifies the understanding of the theory [23].
Validity:	Relates to being valid and offering an accurate representation of the study conducted [25].
Practicality:	Relates to providing a conceptual framework for practice, education and research and being of use to the profession [24, 25].

Adapted from (Chinn & Kramer [27]; Meleis [28]; Parse [29]; Schwaninger & Groesser [30])

**Table 2:** Model validation

Model validation (*N* = 4)				
Criteria	Accepted	Accepted with minimum modification	Not accepted	Comments
Importance	4 (100 %)	0 (0 %)	0 (0 %)	Very relevant for nursing
Precision and clarity	3 (75 %0	1 (25 %)	0 (0 %)	Grammar
Parsimony and simplicity	4 (100 %)	0 (0 %)	0 (0 %)	
Comprehensiveness	4 (100 %)	0 (0 %)	0 (0 %)	
Operationality	4 (100 %)	0 (0 %)	0 (0 %)	
Logical structure	3 (75 %)	1 (25 %)	0 (0 %)	Change visual presentation to horizontal form vertical
Validity	4 (100 %)	0 (0 %)	0 (0 %)	
Practicality	4 (100 %)	0 (0 %)	0 (0 %)	

Adapted from (Chinn & Kramer [27]; Meleis [28]; Parse [29]; Schwaninger & Groesser [30])

All participants indicated that the model was important and significant for nursing. The majority found it clear and precise; although one participant recommended minor grammar and language modifications. The participants found the model simple to understand and uncomplicated. They indicated that it was broad and covered a wide area of interest with regards to nurses’ participation in health policy. They regarded it as specific enough to be operationalized. Most of the expert panelists found the visual diagram clear and simple to understand. One suggested revising the picture to present a horizontal orientation and this was incorporated in the final illustration. The expert panelists indicated that the model was valid and an accurate representation of the findings of the study in which they had participated. Moreover, they indicated that it appeared to provide practical guidance to participation in health policy development.

## Description of the model

**Fig. 1:**
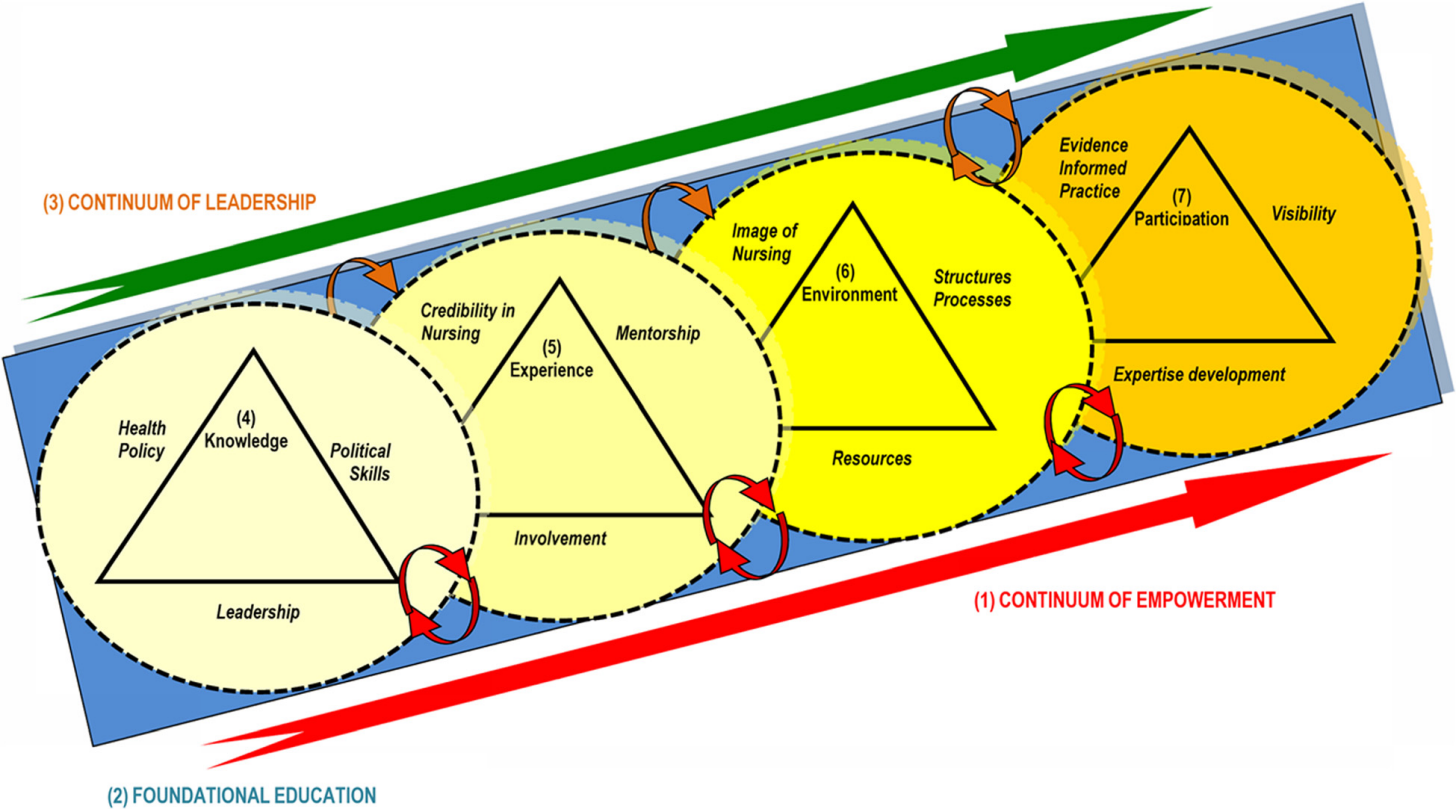
Visual presentation of the “Empowerment model for nurse leaders’ participation in health policy development”

The context of the model, its structure, key concepts and relationships between the concepts are described (see Fig. 1). The model is contextualized within the East African nursing perspective.

### The context of the model

The model provides a framework to support nurses and nurse leaders to enhance their participation in health policy development activities. The model can be used by nurse leaders, nurses, policy makers and nurse educators for this purpose and could be employed for the development of a career pathway regarding the role of a nurse policy maker. The model can be applied by national nurse leaders to mentor and support other nurses in their development in health policy activities. The model offers flexibility in that it can be utilized in its entirety or in part, depending on the self-assessed appropriateness of the individual nurse or user.

### Structure of the model

The model incorporates seven major concepts. The blue base below the circles, the large red and green arrows indicate foundational education, empowerment and leadership. The four circular shapes symbolize knowledge, experience, environment and participation.

The blue block at the base indicates that nurse leaders involved in health policy development must as a minimum have an undergraduate degree as a foundation for such participation. The ascending large green arrow depicts leadership development as it pertains to enhancing nurse leaders’ participation and expertise in health policy development. The little curved arrows on the top of the diagram indicate movement through stages in the continuum. Each stage influences the level of empowerment in the following stage. The ascending large red arrow indicates a developmental empowerment process to participate in the devising of health policy: on the left there is limited empowerment, moving towards greater empowerment on the right, representing dynamic movement. The small red arrows at the bottom suggest a mutual relationship of ‘to be empowered’ and ‘being empowering’. The ascending arrangement of the circular shapes indicates growing empowerment. These shapes are used to indicate an open-ended potential for the growth and expansion of the concepts. The dotted outlines indicate external forces that may influence involvement and may vary significantly from situation to situation. The overlapping of the circles indicates that the concepts are interrelated. The gradual darkening of the colour yellow depicts growing empowerment towards participation in health policy development activities.

### Concepts and relationships inherent in the model

There are seven main concepts proposed in the model (see Fig. 1). Three are fundamental to nurse leaders’ participation in health policy development: foundational education of undergraduate degree, leadership development and continuum of empowerment (see Fig. 1).

Additionally, there are four concepts in the model that exist as stages of empowerment; each builds on the previous stage.

These are: knowledge, experience, environment and participation.

#### Continuum of empowerment (1)

The model illustrates a continuum of growth in terms of empowerment towards participation in health policy development, indicated by a straight red arrow at the base. Increasing empowerment occurs with progression through each stage of the model. ‘To be empowered’ and ‘being empowering’ are the two principles of empowerment that are interactive in the model. Nurse leaders should be supported “to be empowered” to participate in health policy development and should support others to participate by “being empowering”. The little curved red arrows depict this. A reciprocal relationship of giving and receiving mentorship is illustrated with regards to growth in the health policy development field at every stage. Nursing influence in health policy can only be sustained if nurse leaders are supported and if they support and mentor others. Empowerment can be facilitated through: foundational education, leadership, acquiring knowledgeable, gaining experience, creating an enabling environment and ongoing participation.

#### Foundational education (2)

The model demonstrates that foundational education at degree level is important for nurses’ participation in health policy. This is supported by the study findings, specifically consensus that “Nurse Leaders must have at least a university degree-level of education e.g., BScN” with 91 % consensus in both rounds. Furthermore, the findings revealed that the majority of the expert panelists held at minimum an undergraduate degree. Education might provide opportunities and access to health policy development positions.

Degree education facilitates attainment of a level of knowledge and skills which contribute towards the intellectual processes necessary to participate in policy development in a meaningful way. A degree in nursing would place nurse leaders in policy development on a par with other professionals such as pharmacists and medical doctors who are involved in health policy development. Nurses will then share the same educational status, which will accord credibility to their voice [30].

#### Continuum of leadership (2)

Leadership is considered a key component in the capacity of the nurse leader to participate in this arena. The model illustrates a continuum of leadership development which is vital for participation, represented by straight ascending red arrow. This is supported by the study findings, notably consensus that Nurse Leaders must have transformational leadership attributes-being able to influence, being visionary and inspiring a shared vision” with 100 % consensus for both rounds. Essential leadership attributes in the context of this model are those that enable nurse leaders to exert influence in health policy development processes and activities with regards to health and nursing concerns. Leadership attributes can be learnt through basic educational institutions and need to be nurtured through each individual’s career progression. Kunaviktikul et al. [31] study indicates that leadership among nurses would facilitate participation in health policy development activity.

#### Knowledge (4)

Being knowledgeable is a key component and provides the basis of support for nurse leaders towards empowerment in health policy development. Being knowledgeable can equip nurses with the confidence that would facilitate their participation. Being knowledgeable supports other stages of empowerment which include: leadership development; gaining experience, creating an enabling environment and ongoing participation. The knowledge necessary for participation in health policy development can be acquired through tertiary institutions during basic, post basic, masters and doctoral education. Research indicates that acquiring knowledge does change nurses’ policy involvement activity [32]. Three main areas of knowledge required to facilitate participation include: health policy, political skills and leadership.

##### Health policy

This refers to guidelines, directives or principles pertaining to the health sector that govern the action or inaction that influence the health of the population. The study findings indicated consensus that, “Nurse Leaders must be knowledgeable and skilled in the health policy development activities at all levels”, with agreement at 96–100 % among participants. Nurses’ understanding of health policy would create motivation to become involved and should also clarify their roles with regard to participation of this kind [33].

##### Political skills

This refers to being politically astute and being able to lobby with policy makers on issues of concern to the nursing profession. Study findings revealed consensus among the expert panelists that, “Nurse leaders must be politically astute-able to lobby with policy makers and influence health policy of concern to nursing profession”, with 92–100 % consensus. As nurses become politically astute they gain the confidence to lobby with policy makers on issues of concern to the nursing profession. Similarly, Kunaviktikul et al. [31] study indicates that improvement in political skills would enhance involvement in policy activities.

##### Leadership attributes

This refers to being part of and able to influence the health policy making. The study findings indicate that there was agreement among the expert panelists on the leadership attributes that are essential for participation in this arena. Key leadership attributes identified in the study included: influence; transformational attributes and political astuteness; communicate effectively; build relationships; feel empowered and having professional credibility [20]. Leadership attributes can be acquired through tertiary educational institutions which play a key role in facilitating and equipping nurse leaders with education that prepares them to be effective leaders. Furthermore, leadership development occurs through experience, is supported in a conducive environment and is demonstrated and developed by participation in the health policy development process.

#### Experience (5)

This refers to the acquisition of specific experience related to health policy development, reflecting an opportunity to put learned knowledge and skills into practice. Nurses must be nurtured from the early stages of their careers to gain confidence in participation in health policy activities. Furthermore, opportunities should be available at all stages of their career. The study findings revealed consensus that, “Nurse leaders must have experience and exposure to health policy development process” and “Nurse leaders must have opportunities to participate in forums where policies are formulated by policy makers” with 96 %–100 % consensus. The findings revealed factors that could facilitate gaining appropriate experience: professional credibility, involvement and mentorship.

##### Credibility in nursing

This refers to nurse leaders acquiring competence in practical nursing at organizational or community levels. There was 100 % consensus about this area, a finding particularly noted given that a majority of the nurse leaders reported more than 15 years of experience in nursing. Their extensive nursing experience potentially enabled them to gain an understanding of issues of concern to nursing that are related to health policy. Practical nursing experience would inform and validate nurses’ policy related input and in turn would accord credibility to nurse leaders’ voices in health policy.

##### Involvement

This refers to being part of and involved in health policy development activities at the institutional level and in professional nursing organizations. There was consensus that, “Nurse leaders must have opportunities to be included by policy makers at every stage of the health policy development process” with agreement between 96–100 %. Furthermore, nurse leaders indicated that they can become involved in health policy development through: nursing organizations, position(s) held and on an individual basis. Through inclusive work environments and strong professional associations, nurse leaders will gain access to and opportunity to access networks, where they can share and gain experiences and concerns related to health policy.

##### Mentorship

refers to a mutual relationship in which a more experienced person in that field acts as guide and role model to a less experienced person. A mentor provides support in terms of knowledge, advice, and guidance towards achieving greater competence [33]. There was consensus among the nurse leaders that “Nurse Leaders must receive supportive mentorship from leaders who have been involved in and have actively participated in health policy development”. Furthermore there was consensus that, mentorship and support encompass having mentors, accepting and seeking mentorship and providing mentorship with consensus between 96 %–100 %. It is understood that it is the nurse leaders’ responsibility to seek these opportunities and the responsibility of the professional associations and work institutions to facilitate them.

#### Environment (6)

Nurse leaders must seek to create an enabling environment for their participation and legitimate access to involvement in health policy development activities. An enabling environment includes having a positive image of nursing, as well as having supportive structures, processes and resources. The findings of the study indicate that there were significant challenges that nurse leaders encountered as they attempted to participate in policy development processes. Challenges included: lack of opportunity to participate, prohibitive structures and processes, the negative public image of nursing and lack of resources. Phaladze’s [4] study on the role of nurses in HIV/AIDs policy process also found the image and status of nursing to be a barrier to inclusion in the policy development process.

It is envisioned that nurses should gain knowledge and skills pertaining to participation in health policy development through educational institutions. Furthermore, experience within work institutions and professional organizations should facilitate the application of such knowledge and skills as well as equip nurses to influence the creation of an enabling environment. Influencing the environment could be facilitated through positions held, professional organizations, educational institutions, networking and unity of purpose within the nursing profession.

The concepts of environment and participation are interdependent and each mutually influencing. An environment that is not inclusive will make participation in the process difficult; without participation, it may be difficult to create an inclusive and enabling environment. The assumption is that unless nurse leaders are able to create an environment that is inclusive of nursing input in health policy development they will experience difficulties in gaining access to and being part of the policy development process. The findings revealed factors that could facilitate an enabling environment included influencing the: image of nursing; structures and processes; and resources.

##### Image of nursing

This refers to Nurses being respected, included and heard as equal partners with other stakeholders in the policy development process. There was consensus in the current study that nurse leaders’ potential contribution to the health policy process is not recognized as significant by policy makers. Moreover, there was consensus about concern for lack of opportunity to be involved in the policy development process by the policy makers and that input in policy development must be respected by policy makers, with 70–100 % consensus.

There was 100 % consensus about the strategies that were proposed by the expert panelists to enhance the image of nursing. These included: nurse leaders with ability and the right credentials being nominated to national leadership positions; and their engaging the media and policy makers to change the image of nursing and focusing the health policy agenda on issues where nurses can make a contribution, such as health promotion and disease prevention. Nurse leaders must work towards renewing and rebranding the public image of nursing in policy development to gain respect and achieve equal partnership.

##### Structures and processes

This refers to nurse leaders being proactive in influencing the policy development environment to become inclusive and supportive of their participation in this activity at government levels. There was consensus that: current structures and systems largely exclude nurse leaders; health policies are developed at the national level and then rolled down to other levels for implementation. Furthermore, most policy making appointments are given to doctors while nurses were inadequately represented at policy development levels; other professionals purport to represent nurses and nursing issues at policy forums. Moreover, there is lack of clarity about the process of recruitment with regards to nurses at policy level and nursing representation in policy forum in terms of numbers is inadequate (75–100 %). The findings of the current study are consistent with Dollinger’s [30] report that nurses are not able to influence health policy development as they are not present in large enough numbers, lack of status of the nursing profession and the process is dominated by doctors and the medical model.

Nurse leaders can only understand the processes when they participate at every stage of health policy development. The results of the study indicate that nurse leaders must be able to clearly understand the health policy development process. Therefore, the process must be pluralistic and inclusive of nurses at all stages of policy development. Further, the process must be open to information, ideas, research evidence and input from nurse leaders.

##### Resources

This refers to the ability of nurses to generate funds and resources that will assist the profession in activities related to influencing health policy development. There was consensus in the current study that possessing resources facilitated nurse leaders’ participation in health policy development and the corollary, that nurse leaders’ participation facilitated access to resources (91–100 %). Gaining resources for health policy development activity could be facilitated by national nurses’ associations in creating a branch of nurse leaders who are interested in health policy development and advocating for funding.

#### Participation (7)

Participation is conceptualized as being part of and exerting permanent influence, involvement and making a contribution in the process and content of health policy development [31, 34]. At this stage, nurse leaders will have gained competence in participation through their knowledge and experience. Furthermore, nurse leaders will have acquired proficiency through creating an enabling environment by overcoming barriers and facilitating participation. At this stage, they must demonstrate the skills gained in the preceding stages. The key components related to participation include: Expertise development, evidence informed practice and visibility.

##### Expertise development (policy making)

This refers to developing proficiency in policy development activities. The findings of the study indicated that although the majority of expert panelists reported extensive experience in nursing practice, they had limited experience in their current position. A majority of participants reported having up to 5 years of experience related to policy making in their current position. Furthermore, the apparent turnover of nurse leaders might be robbing nursing of expertise and mentorship of future nurse policy makers [6]. Nursing expertise in health policy development can be created if nurse leaders are involved in policy development on a regular basis over a period of time. This would constitute a career pathway for nurses who are potentially interested in furthering their careers in health policy development and being nurse policy makers.

Nurse leader participation in the policy development process, ensuring that nursing concerns are included in the agenda and taking a lead in developing health policy. Nurse leaders need to utilize their competence in health policy development, political skills and leadership attributes to achieve the goals of the nursing health policy agenda. The findings of the study revealed key factors which include: taking a leadership role in the development of health policies that can improve the health of communities and ensure provision of quality health care; nurse leaders must engage policy makers to ensure a bottom-up and top-down approach during the entire policy development process. Furthermore, they must be able to ensure that the health policy agenda is not dominated by medical and curative issues and must be able to focus the health policy agenda around health, which includes health promotion and disease prevention. Nurse leaders should utilize professional associations to support taking a leadership role in the development of health policies that can improve the health of communities and ensure provision of quality health care.

##### Evidence informed practice

This refers to nurses being able to use a research and evidence base to support and inform their proposals related to health policy development. There was consensus among the expert panelists that research, analytical skills and critical thinking skills are necessary to facilitate their participation in the policy development process. Articulating nurses’ position on issues utilizing research may enhance nurses’ image, visibility and credibility among policy makers and peers.

##### Visibility

This refers to being prominent in the policy arena, which could be achieved by actively participating in the policy development process and ensuring that nurses’ voices are heard. Nurse leaders’ participation in health policy could be enhanced if there were greater numbers of them involved at the policy development level: the process must be inclusive and be open to ideas, suggestions and input from nurse leaders who are and desire to be part of the process. Having a critical mass of nurses involved in and familiar with policy development is believed to contribute to influential, informed and united voting on policy matters. In the current study, just over half of the expert panelists participated in health policy implementation [6]. Their participation decreased at other stages such as the problem identification and agenda setting stage. Furthermore, there is need to proactively manage as well as utilize the media to enhance the image of nursing; media can highlight issues of concern related to policy, nursing and health care. In the current study, there was consensus among the expert panelists that it is necessary for nurse leaders to engage the media (91–100 %). The media can be utilized to engage policy makers. Nurses can make use of the media to facilitate creating enabling structures and processes that ensure their input in health policy is included. This also will contribute towards altering the image of nursing, enhancing its visibility and portraying nurses as astute policy makers.

## Discussion and Recommendations

The proposed empowerment model for nurse leaders’ participation in health policy development was developed from the findings of a Delphi study with nurses in current leadership positions. The model presents seven main concepts: continuum of empowerment, foundational education, leadership, knowledge, experience, environment and participation. Basic nursing education for nurse leaders to prepare them to participate in policy development needs to be at the degree level. Nurse leaders’ abilities, as effective leaders, will facilitate their participation in the process. As nurse leaders acquire proficiency in health policy development activities through the various stages they can be empowered. Continuing education is the foundation for such empowerment. It is envisioned that if an education is received that prepares nurses with appropriate knowledge and skills and exposure in health policy, political activism and leadership may generate interest and motivation in nurses to be part of the process and understand their role in health policy development beyond clinical practice. If their basic education includes health policy related knowledge, it is likely that a greater number of them may be interested in devising such policy.

Experience and involvement in health policy development at institutional level and through nursing professional associations will further consolidate the knowledge and skills needed. Nurses will need to be proactive in creating an enabling environment which will enhance their participation in health policy development. Such participation will engender expertise and nursing visibility in the health policy development process.

The empowerment model presented in this paper has been presented as conceptualized by the author. The model needs to be tested broadly and validated further widely within the nursing community in East Africa. The model is based on the results of a Delphi study and utilized a purposive sample of experts, those who participated in the study may or may not represent the broader views of the nurse leaders’.

## Conclusion

There is a window of opportunity for nurses and nurse leaders’ to enhance their participation in health policy development activity. This model provides a framework for facilitating participation in health policy making. Nurses need to be strategic in ensuring that they place themselves and others on the forefront of the policy development arena. The proposed empowerment model suggests proactive and strategic involvement of nurses and nurse leaders in health policy development activities. The model can be utilized across the spectrum of nursing that includes: research; practice; leadership and education.

## Additional file

Additional file 1:
**Third round questionnaire.**
