# Supplementary material for: Empowerment model for nurse leaders’ participation in health policy development: an east African perspective
Source: BMC Nurs. 2015 May 13;14:31. doi: 10.1186/s12912-015-0078-6 (PMC4879743; doi:10.1186/s12912-015-0078-6)
Supplement: Additional file 1: — Third round questionnaire. [file 12912_2015_78_MOESM1_ESM.docx]

**Third Round - Questionnaire**

Please answer all the questions. Mark the option that **most closely** matches your opinion with a cross **[X].**  Please express your opinion by using the key below:

Strongly Agree = **[SA]** Agree = **[A]** Undecided = **[U]** Disagree = **[D]** Strongly Disagree = **[SD]**

**SECTION - A**

**Nurse Leader Characteristics**

1. Leadership attributes that are **essential** for influencing health policy development

| **Item** | **List of factors that influence nurse leaders participation in health policy development**  *(The mean (M), standard deviation (SD) and percentage agreement (PA) of the previous round have been indicated in the brackets)* | **Please indicate your opinion** | | | | |  |
| --- | --- | --- | --- | --- | --- | --- | --- |
|  |  | **SA** | **A** | **U** | **D** | **SD** | **Comments** |
| **1.1.** | Nurse leaders must have transformational leadership attributes - being able to influence, being visionary and inspiring a shared vision *(M=1.25, SD=0.53, PA=100%)* |  |  |  |  |  |  |
| **1.2.** | Nurse leaders must be politically astute - able to lobby with policy makers and influence health policy of concern to nursing profession *(M=1.46, SD=0.66, PA=100%)* |  |  |  |  |  |  |
| **1.3.** | Political skills include: | | | | | | |
|  | 1. Being knowledgeable about the health issues of concern to nursing which are influenced by health policy |  |  |  |  |  |  |
|  | 1. Identify people and build relationships with individuals dealing with your issue of interest at ministry of health level |  |  |  |  |  |  |
|  | 1. Contact policy makers dealing with your issue of interest |  |  |  |  |  |  |
|  | 1. Write to policy makers dealing with your issue of interest – expressing your opinion |  |  |  |  |  |  |
|  | 1. Build coalitions – with groups that have similar interests as nursing e.g. Heart Association to influence reduction in smoking and cardiac diseases |  |  |  |  |  |  |
|  | 1. Be willing to testify to policy makers on issues of concern to nursing profession |  |  |  |  |  |  |
| **1.4.** | Nurse leaders must be effective communicators who is able to articulate and disseminate health policy related issues - listening, speaking, writing *(M=1.21, SD=0.42, PA=100%)* |  |  |  |  |  |  |
| **1.5.** | Nurse leaders must have the ability to clearly articulate health issues of concern to nursing at policy development forums/arena *(M=1.21, SD=0.42, PA=100%)* |  |  |  |  |  |  |
| **1.6.** | Some of the articulation skills nurse leaders must have include: | | | | | | |
|  | 1. Being able to communicate effectively with colleagues in senior and junior positions |  |  |  |  |  |  |
|  | 1. Being able to communicate in the right medium e.g. in person, on the phone, e-mail and media |  |  |  |  |  |  |
|  | 1. Being able to craft and deliver clear messages – e.g. nursing position on proposed health policy |  |  |  |  |  |  |
| **1.7.** | Nurse leaders must have good (effective) interpersonal skills *(M=1.38, SD=0.50, PA=100%)* |  |  |  |  |  |  |
| **1.8.** | Nurse leaders must be able to cultivate cordial working relationships with colleagues and others within and outside the profession, in junior and senior positions *(M=1.42, SD=0.50, PA=100%)* |  |  |  |  |  |  |
| **1.9.** | Nurse leaders must be effective in collaborating and cooperating within and outside the profession *(M=1.33, SD=0.48, PA=100%)* |  |  |  |  |  |  |
| **1.10.** | Nurse leaders must be team players *(M=1.33, D=0.48PA=100%)* |  |  |  |  |  |  |
| **1.11.** | Nurse leaders must have respect for others *(M= 1.48, SD=0.67, PA=100%)* |  |  |  |  |  |  |
| **1.12.** | Nurse leaders must have negotiation skills that generate win-win solutions *(M= 1.21, SD=0.42, PA=100%)* |  |  |  |  |  |  |
| **1.13.** | Nurse leaders must be proactive and take initiative to formulate strategies of being involved at each stage of the policy development process *(M=1.33, SD=0.48, PA=100%)* |  |  |  |  |  |  |
| **1.17.** | Nurse leaders must be creative *(M= 1.26, SD=0.54, PA=100%)* |  |  |  |  |  |  |
| **1.14.** | Nurse leaders must be motivated to participate in health policy development *(M=1.46, SD=0.59, PA=100%)* |  |  |  |  |  |  |
| **1.15.** | Nurse leaders must have personal confidence through encouragement and a feeling of empowerment *(M=1.29, SD=0.46, PA=100%)* |  |  |  |  |  |  |
| **1.16.** | Nurse leaders must be courageous in articulating health issues of concern to nursing *(M=1.29, SD=0.46, PA=100%)* |  |  |  |  |  |  |
| **1.18.** | Nurse leaders must have management skills – planning, organizing, supervising and evaluating *(M= 1.25, SD=0.53, PA=100%)* |  |  |  |  |  |  |
| **1.19.** | Nurse leaders must be knowledgeable and competent in nursing *(M=1.58, SD=0.97, PA=96%)* |  |  |  |  |  |  |
| **1.20.** | Nurse leaders must be assertive in raising nursing concerns related to health care to policy makers *(M=1.38, SD=0.77, PA=96%)* |  |  |  |  |  |  |
| **1.21.** | Nurse leaders must have critical thinking and problems solving skills through nurse leaders education *(M=1.33, SD=0.70, PA=95.8%)* |  |  |  |  |  |  |

**Nurse Leader Characteristics**

1. **Knowledge and skills that are essential for nurse leaders to influence health policy development**

|  | | **SA** | **A** | **U** | **D** | **SD** | **Comments** |
| --- | --- | --- | --- | --- | --- | --- | --- |
| **2.1.** | Nurse leaders must be knowledgeable and skilled in the health policy development activities at all levels *(M=1.42, SD=0.78, PA=95.7%)* |  |  |  |  |  |  |
| **2.2.** | Nurse leaders must have at least a university degree - level of education e.g. BScN *(M=1.63, SD=0.97, PA=90.9%)* |  |  |  |  |  |  |
| **2.3.** | Content related to health policy development must be included in the basic nursing education *(M=1.54, SD=0.66, PA=100%)* |  |  |  |  |  |  |
| **2.4.** | The content in the basic nursing programmes may include: | | | | | | |
|  | 1. Types of policy: public policy, health policy, social policy |  |  |  |  |  |  |
|  | 1. Theories and models of policy making |  |  |  |  |  |  |
|  | 1. Policy development process |  |  |  |  |  |  |
|  | 1. Policy making environment: social, political and economic influences |  |  |  |  |  |  |
|  | 1. Legislative process: district, province, national |  |  |  |  |  |  |
|  | 1. Influencing policy: roles and responsibilities of nurses, strategies to influence policy |  |  |  |  |  |  |
|  | 1. Analyze health policy and political issues |  |  |  |  |  |  |
| **2. 5.** | Nurse leaders must have experience and exposure to health policy development process *(M=1.67, SD=0.76, PA=95.7%)* |  |  |  |  |  |  |
| **2.6.** | Nurse leaders experience and exposure to policy development may include: | | | | | | |
|  | 1. Identifying policy makers and legislators who represent nursing in the community |  |  |  |  |  |  |
|  | 1. Understanding policy makers interests and commitment to health-related issues of concern to nurses |  |  |  |  |  |  |
|  | 1. Analyzing nursing concerns or health issues that can be addressed through policy intervention/reform |  |  |  |  |  |  |
|  | 1. Making presentations, that are evidence based, to policy makers and testifying at legislative hearings |  |  |  |  |  |  |
| **2.7.** | Nurse leaders must have role models through whom they can learn to participate in the health policy development process e.g. directors of medical services who are involved in health policy development *(M=1.42, SD=0.72, PA=96%)* |  |  |  |  |  |  |
| **2.8.** | Nurse leaders must have supportive mentorship from leaders who have been involved in and have actively participated in health policy development *(M=1.33, SD=0.49, PA=100%)* |  |  |  |  |  |  |
| **2.9.** | Supportive mentorship for nurse leaders entails: | | | | | | |
|  | 1. Accepting and seeking mentorship from nurses who have more experience in influencing health policy (expert – novice mentorship) |  |  |  |  |  |  |
|  | 1. Having mentors who inspire, guide, advise and model behavior while they participate in influencing health policy |  |  |  |  |  |  |
|  | 1. Being mentors to nurses with less experience in influencing health policy (peer – peer mentorship) |  |  |  |  |  |  |
| **2.10.** | Nurse leaders need to have networks for support and to share experiences on policy related issues (e.g. national nurses association – intensive care nurses chapter) *(M=1.42, SD=0.50, PA=100%)* |  |  |  |  |  |  |
| **2.11.** | Nurse leaders should develop networks for sharing information, and feedback with: | | | | | | |
|  | 1. Colleagues who have less experience than you |  |  |  |  |  |  |
|  | 1. Colleagues who have equal experience as you |  |  |  |  |  |  |
|  | 1. Colleagues who have more experience than you |  |  |  |  |  |  |
| **2.13.** | Nurse leaders must have opportunities to be included at every stage of the health policy development process by policy makers *(M=1.29, SD=0.86, PA=95.8%)* |  |  |  |  |  |  |
| **2.14.** | Stages of health policy development include: | | | | | | |
|  | 1. Problem identification and agenda setting |  |  |  |  |  |  |
|  | 1. Health policy formulation |  |  |  |  |  |  |
|  | 1. Health policy implementation |  |  |  |  |  |  |
|  | 1. Health policy evaluation |  |  |  |  |  |  |
| **2.15.** | Nurse leaders should be skilled at seeking opportunity for influencing health policy development at: | | | | | | |
|  | 1. Workplace e.g. hospitals |  |  |  |  |  |  |
|  | 1. Community – e.g. the village/constituency they live in |  |  |  |  |  |  |
|  | 1. Professional associations – national nurses associations |  |  |  |  |  |  |
|  | 1. Government – ministry of health |  |  |  |  |  |  |
| **2.16.** | Nurse leaders must participate actively in the entire policy making process when given the opportunity to participate *(M=1.21, SD=0.42, PA=100%)* |  |  |  |  |  |  |
| **2.17.** | Active participation includes: | | | | | | |
|  | 1. Articulating issues of concern to nursing |  |  |  |  |  |  |
|  | 1. Ensuring that nursing is positioned in the mainstream of health policy development to acquire power and influence |  |  |  |  |  |  |
|  | 1. being visible |  |  |  |  |  |  |
|  | 1. being accessible |  |  |  |  |  |  |
| **2.18.** | Nurse leaders must have the ability to engage the media to change the image of nursing *(M=1.71 , SD=0.91, PA=91% )* |  |  |  |  |  |  |
| **2.19.** | Media management skills include: | | | | | | |
|  | 1. Articulating clearly issues of concern to nursing |  |  |  |  |  |  |
|  | 1. Be proactive in communicating with the media on health and nursing concerns |  |  |  |  |  |  |
|  | 1. Responding to media releases related to health and nursing concerns |  |  |  |  |  |  |
|  | 1. Using the media as a medium for nurses to inform policy makers of nurses’ contribution to health care |  |  |  |  |  |  |
| **2.22.** | Nurse leaders must engage policy makers to ensure a bottom up and top down approach during the entire policy development process *(M=1.54, SD=0.72, PA=100%)* |  |  |  |  |  |  |
| **2.20.** | Nurse leaders input in policy development must be respected by policy makers *(M=1.38, SD=0.50, PA=100%)* |  |  |  |  |  |  |
| **2.21.** | Nurse leaders with the ability (right credentials) to influence health policy should be nominated to national leadership positions e.g. Director of Nursing Services *(M=1.25, SD=0.53, PA=100%)* |  |  |  |  |  |  |
| **2.23.** | Nurse leaders must be able to focus the health policy agenda around health which includes health promotion and disease prevention |  |  |  |  |  |  |
| **2.24.** | Nurse leaders must be able to ensure that the health policy agenda is not dominated by medical and curative issues |  |  |  |  |  |  |
| **2.25.** | Nurse leaders must have research skills and analytical skills to inform the health policy agenda with evidence *(M=1.33, SD=0.57, PA=100%)* |  |  |  |  |  |  |
| **2.26.** | Research skills include: | | | | | | |
|  | 1. Ability to find appropriate evidence |  |  |  |  |  |  |
|  | 1. Ability to analyze the usefulness of evidence |  |  |  |  |  |  |
|  | 1. Having the ability to effectively communicate to and disseminate research findings to policy makers and stakeholders (*M=1.21, SD=0.42, PA=100%)* |  |  |  |  |  |  |
|  | 1. Ability to use research evidence when advocating and influencing health policy development with regards to nursing concerns |  |  |  |  |  |  |
| **2.27.** | Nurse leaders must have business and financial skills to ensure that they are able to secure financial resources for policy development process |  |  |  |  |  |  |
| **2.28.** | Nurse leaders must be able to mobilise funds to finance policy making activities *(M=1.79, SD=0.72, PA=95.7%)* |  |  |  |  |  |  |
| **2.29.** | Nurse leaders must be part of and actively participate in national nurses associations *(M= 1.71, SD=0.75, PA=100%)* |  |  |  |  |  |  |
| **2.30.** | Some activities of the national nursing organization may include: | | | | | | |
|  | 1. Identifying issues of concern to nurses and health care |  |  |  |  |  |  |
|  | 1. Drawing the attention of the public to issues of concern to nursing and health care |  |  |  |  |  |  |
|  | 1. Taking a leadership role in the development of health policies that can improve the health of communities and ensure provision of quality health care |  |  |  |  |  |  |
|  | 1. Gaining the collective participation and support of nurses |  |  |  |  |  |  |
| **2.12.** | Nurses leaders should be united as a profession and articulate issues of concern to nursing profession and health services through nurses professional organisation *(M=1.42, SD=0.78, PA=96%)* |  |  |  |  |  |  |
| **2.31.** | Nurse leaders should be supported by international organizations e.g. WHO and ICN that can develop and strengthen the role of the nurses in health policy development *(M=1.54, SD=0.83, PA=95.5%)* |  |  |  |  |  |  |

**SECTION - B**

**Structures**

1. **Structures that facilitate Nurse Leaders participation in health policy development**

|  | | **SA** | **A** | **U** | **D** | **SD** | **Comments** |
| --- | --- | --- | --- | --- | --- | --- | --- |
| **1.1.** | Having legislature that ensures that national nurse leaders are included in the health policy development process *(M=1.38, SD=0.65, PA=100%)* |  |  |  |  |  |  |
| **1.2.** | National nurse leaders to be included in health policy development include: | | | | | | |
|  | 1. Nurse leaders from National Nurses Association |  |  |  |  |  |  |
|  | 1. Nurse leaders from Ministry of Health (national offices) |  |  |  |  |  |  |
|  | 1. Nurse Leaders represented on Nursing Councils |  |  |  |  |  |  |
|  | 1. Nurse leaders from academic institutions |  |  |  |  |  |  |
| **1.3.** | Nursing must have a directorate of nursing services who is on par with the director of medical services (or equivalent) at the ministry of health or equivalent *(M=1.42, SD= 0.72, PA=100%)* |  |  |  |  |  |  |
| **1.4.** | Nurses must have leadership positions allocated for nurse leaders at policy making levels (affirmative action) *(M=1.46, SD= 0.66,* *PA=100%)* e.g. permanent secretaries, Director of nursing service |  |  |  |  |  |  |
| **1.5.** | Nurse leaders must have opportunities to participate in forums where policies are formulated by policy makers *(M=1.42, SD=0.58, PA=100%)* |  |  |  |  |  |  |
| **1.6.** | Policy makers must enhance the representation (numbers) of nurse leaders at national policy making level *(M=1.75, SD=2.03, PA=95.7%)* |  |  |  |  |  |  |
| **1.7.** | Policy makers must ensure that they have a gender balance (nurse leaders must be proportionate to the percentage of women and men in the nursing profession) at health policy development positions (*M=2.13, SD=0.90, PA=90%)* |  |  |  |  |  |  |
| **1.8.** | Nurse leaders must have resources allocated for their participation in policy development activities e.g. financial, material and human *(M=1.58, SD=0.93, PA=91.3%)* |  |  |  |  |  |  |
| **1.9.** | Nurse leaders must have access to strong nurses associations (National Nurses Association or Union which are allowed an unrestricted voice by legislature) *(M=1.46, SD=0.59, PA=100%)* |  |  |  |  |  |  |

**SECTION - C**

**Policy Development Process**

1. **Processes that facilitate Nurse Leaders participation in health policy development**

**Facilitators - General**

|  | | **SA** | **A** | **U** | **D** | **SD** | **Comments** |
| --- | --- | --- | --- | --- | --- | --- | --- |
| **1.1.** | Nurse leaders must have opportunities to be included at every stage of the health policy development process by policy makers *(M=1.29, SD=0.86, PA=96%)* |  |  |  |  |  |  |
| **1.2.** | For nurse leaders to be able to participate effectively in the health policy development: | | | | | | |
|  | 1. The processes must be clear to nurse leaders |  |  |  |  |  |  |
|  | 1. The processes must be pluralistic and include nurse leaders |  |  |  |  |  |  |
|  | 1. The information related to the operation of the processes must be available to nurse leaders |  |  |  |  |  |  |
|  | 1. The processes must be open to information, ideas, research evidence and input from nurse leaders |  |  |  |  |  |  |

**Facilitators – Problem identification/agenda setting**

|  | | **SA** | **A** | **U** | **D** | **SD** | **Comments** |
| --- | --- | --- | --- | --- | --- | --- | --- |
| **1.3.** | Nurse leaders must be part of the agenda setting and problem identification process *(M=1.29, SD=0.55, PA=100%)* |  |  |  |  |  |  |
| **1.4.** | Nurse leaders must understand the objectives of the policy makers and government, so that they can align nursing proposals within that context (*M=1.29, SD=0.46, PA=100%)* |  |  |  |  |  |  |

**Facilitators - Health policy formulation**

|  | | **SA** | **A** | **U** | **D** | **SD** | **Comments** |
| --- | --- | --- | --- | --- | --- | --- | --- |
| **1.5.** | Nurse leaders must be able to lobby with influential people (policy makers) to support nurse leaders suggestions in terms of the content of the proposed health policy *(M=1.42, SD=0.58, PA=100%)* |  |  |  |  |  |  |

**Facilitators - Health policy implementation**

|  | | **SA** | **A** | **U** | **D** | **SD** | **Comments** |
| --- | --- | --- | --- | --- | --- | --- | --- |
| **1.6.** | Nurse leaders must be part of the policy formulation process so that they understand and are part of the health policy implementation process *(M=1.25, SD=0.44, PA=100%)* |  |  |  |  |  |  |
| **1.7.** | For nurse leaders to participate effectively: | | | | | | |
|  | 1. Policy implementation process must be clear and transparent |  |  |  |  |  |  |
|  | 1. Having the health policies accessible to nurse leaders *(M=1.21, SD=0.42, PA=100%)* |  |  |  |  |  |  |
|  | 1. Nurse leaders must be empowered on health policy implementation *(M=1.21, SD=0.42, PA=100%* |  |  |  |  |  |  |
| **1.8.** | Nurse leaders must be provided with resources like finances, to ensure implementation of health policies *(M=1.50, SD=0.72, PA=100%)* |  |  |  |  |  |  |
| **1.9.** | Nurse leaders must have access to forums to discuss health policy implementation issues e.g. reduction of teenage pregnancy *(M=1.42, SD=0.72, PA=95.8%)* |  |  |  |  |  |  |

**Facilitators - Health policy evaluation**

|  | | **SA** | **A** | **U** | **D** | **SD** | **Comments** |
| --- | --- | --- | --- | --- | --- | --- | --- |
| **1.10.** | Nurse leaders must be involved in formulating policy evaluation tools *(M=1.46, SD=0.51, PA=100%)* |  |  |  |  |  |  |
| **1.11** | Nurse leaders must participate in setting measureable and achievable targets of health policy evaluation *(M=1.38, SD=0.50, PA=100%)* |  |  |  |  |  |  |
| **1.12** | For nurse leaders to participate effectively in the policy evaluation process there must be: | | | | | | |
|  | 1. Clear policy evaluation process and tools developed by policy makers *(M=1.38, SD=0.50, PA=100%)* |  |  |  |  |  |  |
|  | 1. Clarity of the outcomes to be evaluated |  |  |  |  |  |  |
|  | 1. Funds and resources available to evaluate policy *(M=1.50, SD=0.93, PA=95.5%)* |  |  |  |  |  |  |

**SECTION - D**

**Barriers**

1. **Factors that hinder Nurse Leaders participation in health policy development**

|  | | **SA** | **A** | **U** | **D** | **SD** | **Comments** |
| --- | --- | --- | --- | --- | --- | --- | --- |
| **1.1.** | Nurse leaders potential contribution to policy process is not recognized as significant by the policy makers *(M=1.65, SD=0.78, PA=95.5%)* |  |  |  |  |  |  |
| **1.2.** | Nurse leaders input is called upon on ad hoc basis and they are not part of the full policy process *(M=1.54, SD=0.77, PA=100%)* |  |  |  |  |  |  |
| **1.3.** | Lack of opportunity for Nurse leaders to be involved in the whole process of policy development *(M=1.58, SD=0.72, PA=100%)* |  |  |  |  |  |  |
| **1.4.** | Nurse leaders lack opportunity to be involved in policy development at: | | | | | | |
|  | 1. Workplace e.g. hospitals |  |  |  |  |  |  |
|  | 1. Community – e.g. the village they live in |  |  |  |  |  |  |
|  | 1. Professional associations – national nurses associations |  |  |  |  |  |  |
|  | 1. Government – ministry of health |  |  |  |  |  |  |
| **1.5.** | Lack of financial, material and human resources to implement health policy *(M=1.75, SD=1.07, PA=91%)* |  |  |  |  |  |  |
| **1.6.** | Lack of clear monitoring and evaluation of health policy implementation process by policy makers at the ministry of health *(M=1.88, SD=0.99, PA=91%)* |  |  |  |  |  |  |
| **1.7.** | The policy evaluation process is conducted by consultants attached to the relevant ministry (ministry of health) hence nurse leaders are excluded at this stage of the process *(M=1.54, SD=0.93, PA=96%)* |  |  |  |  |  |  |
| **1.8.** | Lack of opportunity for nurse leaders to be involved in the policy evaluation process *(M=1.33, SD=0.48, PA=100%)* |  |  |  |  |  |  |
| **1.9.** | Nurse leaders lack of opportunity to be involved in the policy development process by the policy makers *(M=1.17, SD=0.38, PA=100%)* |  |  |  |  |  |  |
| **1.10.** | Institutional structures and systems are such that they exclude nurse leaders from being part of the policy process *(M=1.58, SD=0.83, PA=95.5%)* e.g. Nurse leaders are in relatively junior positions |  |  |  |  |  |  |
| **1.10.** | Unclear policies of recruitment of nursing leaders at policy level |  |  |  |  |  |  |
| **1.12.** | Health policies are developed at national level and then rolled down to other levels for implementation (M=1.46, SD 0.72, PA=100%) |  |  |  |  |  |  |
| **1.13.** | These levels of health policy implementation include: | | | | | | |
|  | 1. District level |  |  |  |  |  |  |
|  | 1. Provincial level |  |  |  |  |  |  |
|  | 1. National level |  |  |  |  |  |  |
| **1.14.** | Inadequate representation (numbers) of nurse leaders at the policy making forums *(M=1.54, SD=0.83, PA=95.5%)* |  |  |  |  |  |  |
| **1.15.** | Most appointments into policy making positions are given to doctors (*M=1.29, SD=0.55, PA=100%)* |  |  |  |  |  |  |
| **1.16.** | Other health professionals including doctors represent nurses and nursing issues at health policy development forums *(M=1.58, SD=0.78, PA=100%)* as structures are not inclusive of nurse leaders |  |  |  |  |  |  |

Thank you for your support

Nilufa
